# Genome-resolved insight into the reservoir of antibiotic resistance genes in aquatic microbial community

**DOI:** 10.1038/s41598-022-25026-3

**Published:** 2022-12-06

**Authors:** Zahra Goodarzi, Sedigheh Asad, Maliheh Mehrshad

**Affiliations:** 1 Department of Biotechnology, College of Science, University of Tehran, Tehran, Iran; 2 Department of Aquatic Sciences and Assessment, Swedish University of Agricultural Sciences (SLU), Box 7050, 75007 Uppsala, Sweden

**Keywords:** Water microbiology, Antimicrobial resistance

## Abstract

Aquatic microbial communities are an important reservoir of antibiotic resistance genes (ARGs). However, distribution and diversity of different ARG categories in environmental microbes with different ecological strategies is not yet well studied. Despite the potential exposure of the southern part of the Caspian Sea to the release of antibiotics, little is known about its natural resistome profile. We used a combination of Hidden Markov model (HMM), homology alignment and a deep learning approach for comprehensive screening of the diversity and distribution of ARGs in the Caspian Sea metagenomes at genome resolution. Detected ARGs were classified into five antibiotic resistance categories including prevention of access to target (44%), modification/protection of targets (30%), direct modification of antibiotics (22%), stress resistance (3%), and metal resistance (1%). The 102 detected ARG containing metagenome-assembled genomes of the Caspian Sea were dominated by representatives of Acidimicrobiia, Gammaproteobacteria, and Actinobacteria classes. Comparative analysis revealed that the highly abundant, oligotrophic, and genome streamlined representatives of taxa Acidimicrobiia and Actinobacteria modify the antibiotic target via mutation to develop antibiotic resistance rather than carrying extra resistance genes. Our results help with understanding how the encoded resistance categories of each genome are aligned with its ecological strategies.

## Introduction

Antibiotic resistance is a major threat to modern society. Projections indicate that the antimicrobial resistance (AMR) attributable mortality could reach up to 10 million by 2050^1^. Understanding the connections between the human, animal and environmental microbiome (the One Health concept) is critical for tackling AMR crisis as a complex, transboundary, and multifactorial health challenge^2,3^. Thus prevention, surveillance and control of AMR require integrated political and socio-economic actions which require a comprehensive ecological surveillance networks^2,4^.

Despite its adverse effect on human health, AMR is a natural phenomenon^5^. While it is clear that excessive use of antibiotics significantly contributes to the emergence of resistant strains, antibiotic resistance also exists in natural bacteria of pristine ecosystems^6^. Antibiotics and antibiotic resistance genes (ARGs) have been co-evolving in the ecosystems for millions of years^7^ (Mostly fueled by microbe’s continuous competition for resources). In addition to their well-known role, antibiotics and ARGs play other physiological roles in nature^8^. For example, at sub-inhibitory concentrations, antibiotics act as signaling molecules involved in quorum sensing and biofilm formation^9,10^. Some ARGs were originally involved in cellular functions such as virulence, cell homeostasis and intercellular signal trafficking^11,12^, but were selected for the resistance phenotype and got transferred from the environmental reservoirs into commensal and pathogenic bacteria ^8,11^. Following the widespread presence of antibiotics, this transfer occurred very rapid on an evolutionary scale through horizontal gene transfer (HGT) and mobile genetic elements (MGEs)^13^. Environmental microbiome have been shown to serve as potential reservoirs of antibiotic resistance genes primed for exchange with pathogenic bacteria^14^. Nevertheless, the evolution and prevalence of ARGs in environmental microorganisms is poorly understood^7^.

Antibiotics are currently widely used, not just for the treatment of human infections, but also in agriculture^15^, livestock^16^, and aquaculture industries^17^. Discharge of antimicrobials and resistant micro-organisms in waste from healthcare facilities^18^, pharmaceutical manufacturing facilities^19^ and other industries into the environment and mostly to aquatic environments affects the natural ecosystems^20^. This has been shown to accelerate development and transfer of AMR among bacterial populations in clinical and natural environments through selection pressures^21^. This concern is also growing by global warming as it might accelerate the spread of antibiotic resistance^22^.

Meta-omics studies from different natural ecosystems specially aquatic environments such as ocean^23,24^, rivers^25^, lakes^26^ and sea water^27^ have recently detected ARGs and profiled the antibiotic resistome of these ecosystems. These studies reiterate that even natural environments that have not been exposed to high antibiotic concentrations could potentially be a reservoir of ARGs. While recovered ARGs in oceanic ecosystems mainly belong to representatives of Gamma- and Alpha- proteobacteria^23,28^, comparative analysis of ARGs present in different taxa in relation to their ecological strategies is missing. Investigating the environmental reservoirs of ARGs, their presence on horizontally transferable mobile genetic elements (MGEs), taxonomic affiliation of the Antibiotic resistant bacteria (ARBs), and their ecological strategies is critical to assess their contribution to emergence and spread of ARGs as well as future actions to fight resistant infections.

The southern part of the Caspian Sea (bordering with Iran) is increasingly exposed to human caused pollution due to high percentage of organic matter entering the basin via agricultural and aquaculture effluents^29^. Additionally, WHO report on surveillance of antibiotic consumption puts Iran among countries with high-level use of antibiotics^30^ which possibly would further leak into aquatic ecosystems. Yet still robust and comprehensive monitoring programs for this ecosystem are largely missing and we lack a survey for the status of antibiotic pollution in the Caspian Sea. Additionally, Caspian Sea is vulnerable to climate crisis. Because of the sea level decline predicted for the Caspian Sea the more shallow northern part of the sea will disappear and the overall lake ecosystem and its biota are in danger of being adversely affected^31^. Consequently, understanding the ARG reservoir of the Caspian Sea is critical for monitoring and conservation purposes. To this end, we performed genome-resolved metagenomic analyses for ARGs in the deeply sequenced depth profile metagenomes of the Caspian Sea. We applied Hidden Markov model (HMM), homology alignment and a deep learning approach supplemented with manual curation of potential ARGs and classified them into five antibiotic resistance categories. The results of metagenomic ARG surveys, are constrained by the comprehensiveness and quality of the used antimicrobial resistance gene databases^32^. Here we used different databases of protein and nucleotide sequences as well as different approaches to provide a comprehensive genome-resolved view of the Caspian Sea’s resistome. Moreover, we studied these approved ARGs in relation to the ecological strategies of ARBs containing them. Our results show that most of the ARG containing metagenome-assembled genomes (MAGs) are among taxa that are still evading the bound of culture. More interestingly, we see that the streamlined genomes mainly contain ARGs with mutations in the antibiotic target rather than carrying extra genes for antibiotic resistance.

## Results and discussion

### Caspian Sea MAGs characteristics

In this study, we explored the diversity and distribution of ARGs in three metagenomes collected along the depth profile of the brackish Caspian Sea. Binning resulted in 477 metagenome-assembled genomes (MAGs) with completeness ≥ 40% and contaminations ≤ 5%. Only 14 MAGs belonged to domain Archaea and the rest of 463 bacterial MAGs were dominated by Proteobacteria, Bacteroidota, and Actinobacteriota (Overall taxonomic distribution is shown in Supplementary Figure S1).

### Antibiotic resistance gene profile of the Caspian Sea Bacteria

Using six different tools we initially detected in total 259 potential ARGs in 110 MAGs. All predicted genes were further manually checked for conserved domains to confirm the functional predictions. For detected genes that confer resistance to antibiotics due to mutations we manually checked the alignments and report them as potential resistance genes only when they contained the exact mutation as those reported to cause resistance. A total of 82 genes conferring antibiotic resistance due to mutation were initially detected. Multiple sequence alignment together with reference genes confirmed mutation in 56 genes, two parC gene, three murA genes, 31 rpsL genes and 20 rpoB genes (multiple sequence alignments and mutations conferring antibiotic resistance are shown in the Supplementary Figure S2). There are ongoing debates regarding the relevance of detected mutations in annotated genes to exhibiting resistant phenotype in the organism^33–35^. While our additional alignment results confirm the presence of exact mutations for antibiotic resistance via these genes, experimental tests are the ultimate confirmation of the resistant phenotype.

**Figure 1:**
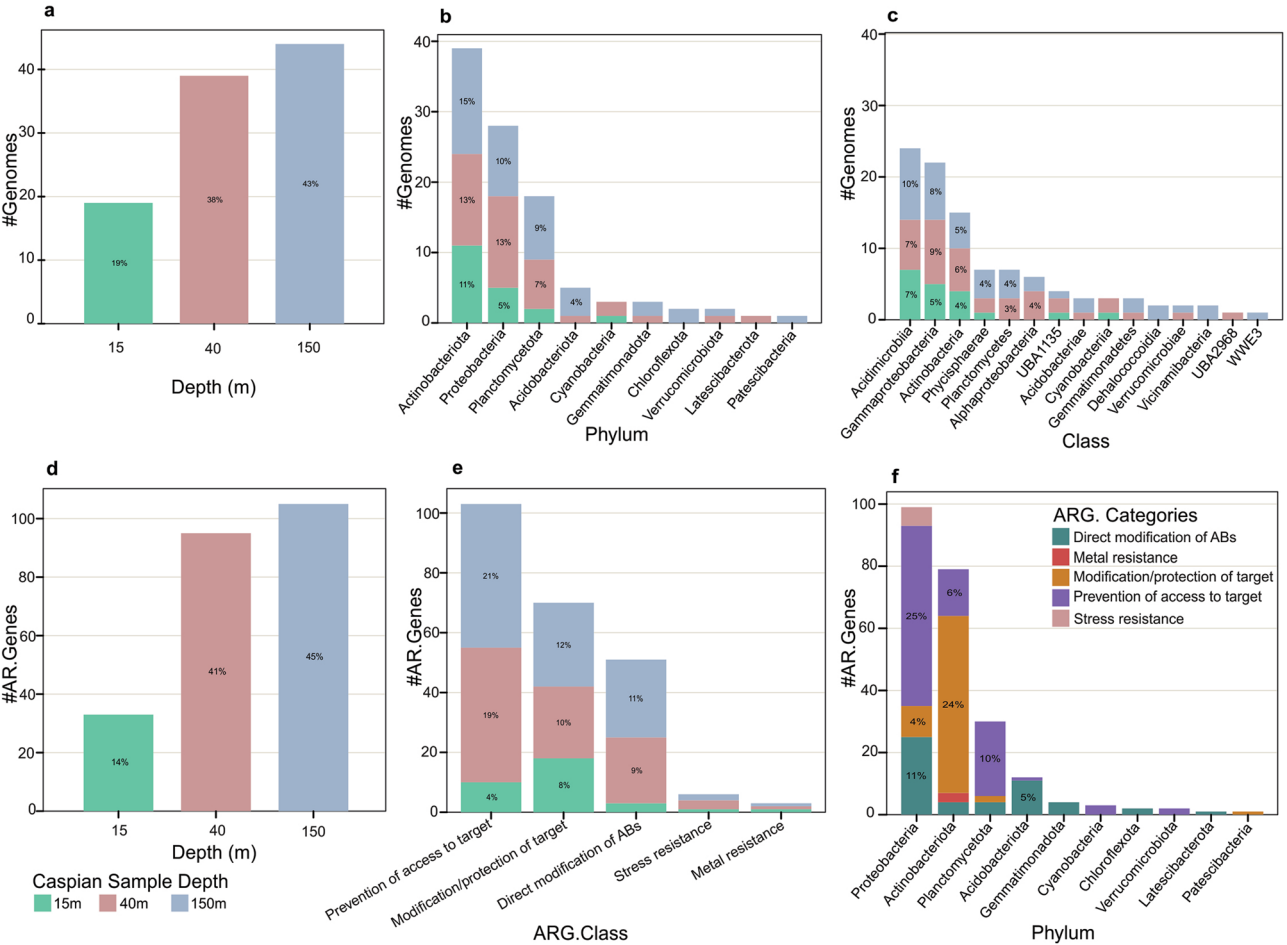
Distribution of ARGs and ARG containing MAGs in the Caspian Sea metagenomes. Distribution of ARG containing MAGs based on depth (**a**), and phylogenetic diversity at the phylum (**b**), and class (**c**) level. Distribution of ARGs in different depth (**d**), abundance of ARGs per ARG categories in different depth (**e**), and abundance of ARG categories based on phylogenetic diversity at the phylum level (**f**).

After this curation step, annotations of 33, 95, and 105 predicted genes was confirmed as putative ARGs in respectively 15, 40, and 150 m depth (Fig. 1d). These ARGs were distributed in 102 bacterial genomes (Fig. 1a). Confirmed ARGs identified via each screening tool are detailed in the Supplementary Table S1. Antibiotic resistant bacteria (ARB) were more diverse in 150 m (13 different classes) and 40 m (12 different classes) depths as compared to the 15 m (6 different classes) depth. In general, a higher phylogenetic diversity was detected in the deeper strata of the Caspian Sea^36^.

**Figure 2:**
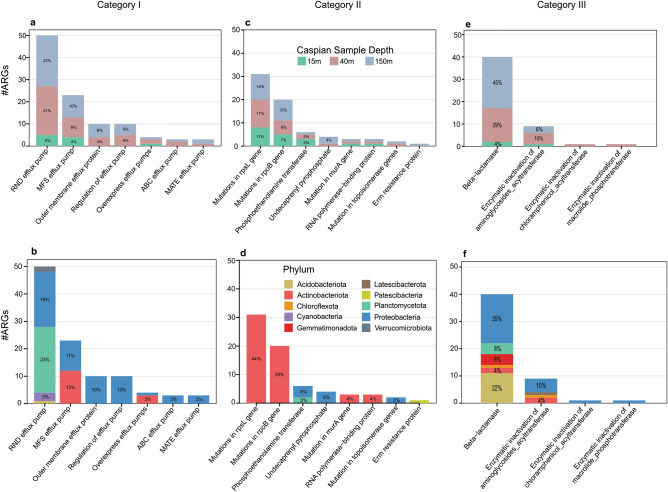
Distribution of ARGs subcategories in Caspian Sea metagenomes based on depth and their phylogenetic diversity at the phylum level. Distribution of ARGs in: (**a**, **b)** category (I) Prevention of access to target, (**c**, **d**) category (II) modification/protection of targets, and (**e**, **f**) category (III) direct modification of antibiotics.

The Caspian ARGs were classified into 5 different antibiotic resistance categories according to their annotated functions: (I) prevention of access to target, (II) modification/protection of targets, (III) direct modification of antibiotics, (IV) stress resistance, and (V) metal resistance (stats of these categories and their subcategories are shown in the Table 1). The most frequently detected category (44%) was prevention of access to target (due to prevalence of antibiotic efflux pumps) followed by, modification and protection of targets (30%) (Fig. 2) and direct modification of antibiotics (22%). The rest of identified ARGs were classified in two categories of stress (6 genes or 3%) and metal (3 genes or 1%) resistance. Categories (I), (II) and (III) ARGs were less prevalent in 15 m depth metagenome of the Caspian Sea (Fig. 1e).

**Table 1:** Distribution of detected Caspian Sea ARGs in different categories and subcategories.

ARG category	ARG subcategory	#ARG
**Prevention of access to target**		103
	ABC efflux pump	3
	MATE efflux pump	3
	MFS efflux pump	23
	Outer membrane efflux protein	10
	Overexpress efflux pumps	4
	Regulation of efflux pump	10
	RND efflux pump	50
**Modification (and protection) of targets**		70
	Erm resistance protein	1
	Mutation in topoisomerase genes	2
	Mutation in murA gene	3
	Mutation in rpoB gene	20
	Mutation in rpsL gene	31
	Phosphoethanolamine transferase	6
	RNA polymerase-binding protein	3
	Undecaprenyl pyrophosphate	4
**Direct modification of antibiotics**		51
	Beta-lactamase	40
	Enzymatic inactivation of aminoglycosides_acyltransferase	9
	Enzymatic inactivation of chloramphenicol_acyltransferase	1
	Enzymatic inactivation of macrolide_phosphotransferase	1
**Stress resistance**		6
**Metal resistance**		3
**Total**		233

We detected six cyclic AMP (cAMP) receptor protein (CRP) genes in the stress resistance category. All of these six stress resistance genes belong to the class Gammaproteobacteria (Fig. 1f) and five of them belong to the family *Pseudohongiellaceae*. CRP, a global transcriptional regulator, contributes to emergence of stress resistance in bacteria through its regulatory role in multiple cellular pathways, such as anti-oxidation and DNA repair pathways. Stress responses play an important role in integron rearrangements, facilitating the antibiotic resistance acquisition and development, and ultimately the emergence of multidrug-resistant bacteria. So, understanding the evolution of bacterial stress responses is critical, since they have a major impact on the evolution of genome plasticity and antibiotic resistance^37^.

A recent study demonstrates the potential contribution of metal resistance genes and plasmidome to the stabilization and persistence of the antibiotic resistome in aquatic environments^38^. We identified three ferritin genes classified in the metal resistance category, in the Caspian Sea MAGs affiliated to genus *Mycolicibacterium*. Ferritin (bfr) is an iron storage protein involved in protection of cells against oxidative stress (iron-mediated oxidative toxicity) and iron overload^39,40^.

**Figure 3:**
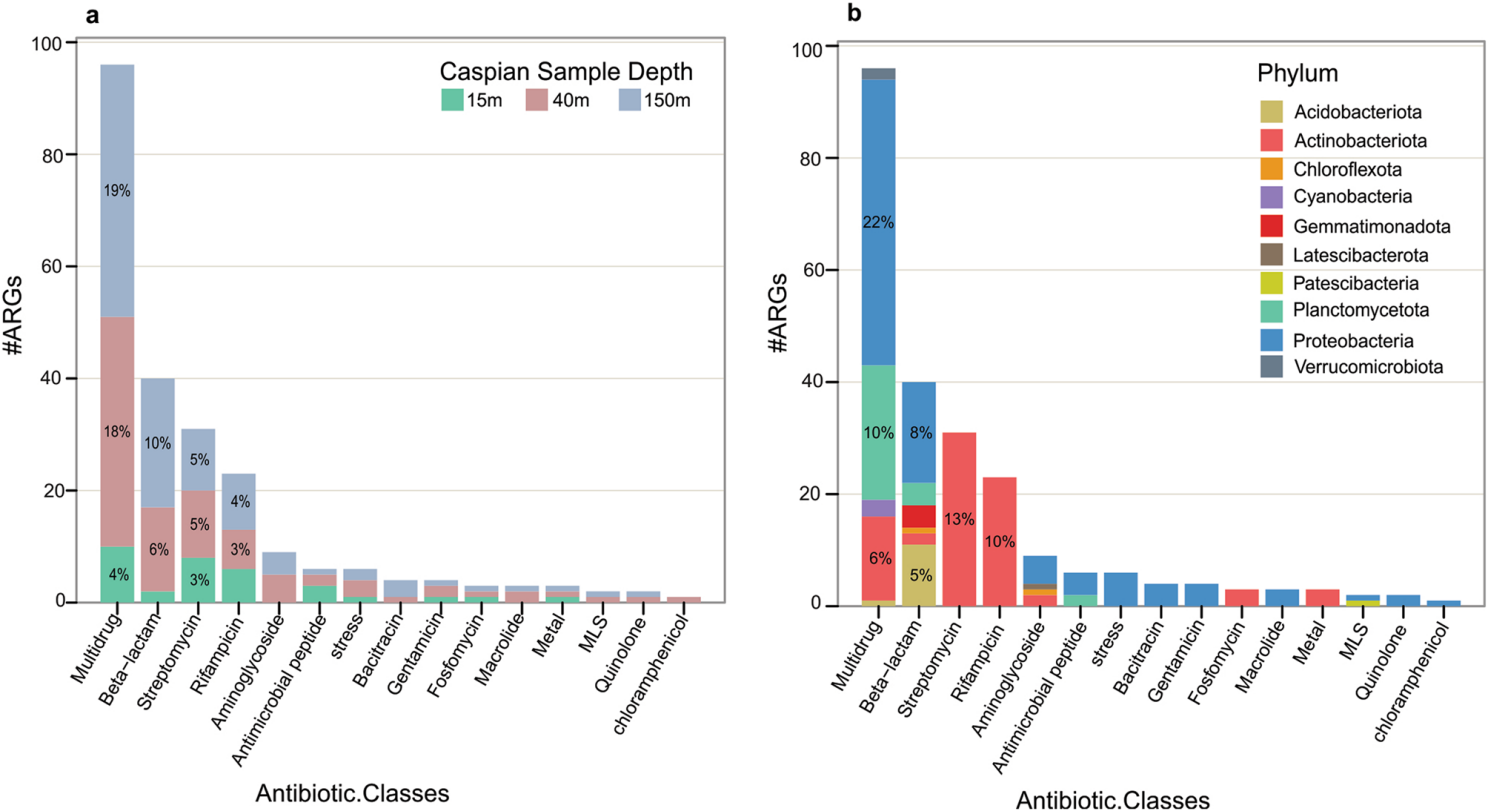
Abundance and distribution of drug classes that the antibiotic resistance genes of the Caspian Sea metagenomes can provide resistance against. Distribution of drug classes based on depth (**a**), and phylogenetic diversity at the phylum level (**b**).

The most frequent subcategory detected in the Caspian Sea MAGs was RND efflux pump (50 ARGs), β-lactamase (40 ARGs), and mutation in rpsL gene (31 ARGs), respectively (Table 1). In category Prevention of access to target, Caspian ARGs are classified into different types of efflux pumps and their regulatory sequences (Fig. 2a,b). Besides, all resistances caused by mutational changes are in category modification/protection of targets (Fig. 2c,d). In the category of direct modification of antibiotics, Caspian ARGs belong to β-lactamases and some transferases (Fig. 2e,f). Many ARGs provide resistant to several classes of antibiotics in bacteria thus majority of the Caspian Sea ARGs belong to the multidrug antibiotic class (Fig. 3). The term "multidrug" here refers to different classes of antibiotics that a certain ARG can offer resistance against. For instant, genes related to efflux pumps can offer resistance to a number of antibiotic classes and hence here they were considered as a part of the multidrug class in Fig. 3. Genes encoding multidrug efflux pumps are evolutionarily ancient elements and are highly conserved^11^. The frequency of the efflux mediated antibiotic resistance in other environments^23,28^ supported that efflux pumps have other physiologically relevant roles such as detoxification of intracellular metabolites, stress response and cell homeostasis in the natural ecosystems^11^. The second antibiotic class that the Caspian Sea ARGs provide resistance to is the β-lactams class. many soil bacteria have been isolated that can grow on β-lactam antibiotics as the sole source of carbon^41,42^. The abundance of β-lactamases in the Caspian Sea MAGs could also be related to other ecological roles of β-lactam.

### Taxonomic distribution of ARG containing genomes

A total of 233 resistance genes were identified from 102 reconstructed MAGs of the Caspian Sea (MAG stats are shown in the Supplementary Table S3). These MAGs were assigned to 10 phyla dominated by Actinobacteriota (79 ARGs in 39 ARBs) and Proteobacteria (99 ARGs in 28 ARBs) (Fig. 1b,f). Identified ARGs were distributed in 15 classes showing the highest abundance in Acidimicrobiia (24 ARBs), Gammaproteobacteria (22 ARBs) and Actinobacteria (15 ARBs) (Fig. 1c). Although Bacteroidota constitutes 18% of reconstructed Caspian Sea MAGs, no resistance gene was detected in MAGs affiliated to this phylum (Supplementary Figure S1). Moreover, 64% of the Caspian Sea ARG containing MAGs had a single ARG, 18% had two ARGs, and rest of them (18%) had multiple ARGs. Multidrug resistant microbes are defined as those with resistance to three or more classes of antibiotics^43^. Multidrug resistant bacteria constitute 18% of Caspian Sea ARG containing MAGs dominated by representatives of Proteobacteria. (Supplementary Figure S7).

The casp40-mb.75 and casp150-mb.119 MAGs contained ARGs belonging to four different groups of resistance genes (Supplementary Figure S4). Both MAGs contain an ARG in the metal resistance group (and no stress resistance gene). These MAGs are taxonomically affiliated to the genus *Mycolicibacterium* and show a higher abundance at 40 and 150 m depths (ca. 1300 TPM) (Supplementary Figure S5). The genus *Mycolicibacterium* comprise a wide range of environmental and pathogenic bacteria that are potential hosts of ARGs and MGEs. This may contribute to their diversity and evolution or even to their success as opportunistic pathogens^44^. Studies conducted in Japan suggest that livestock could acquire *Mycolicibacterium peregrinum* from their environment^45^. Presence of *Mycolicibacterium* representatives containing a set of ARGs in the natural environment could be a reservoir of genes for potential development of resistance in pathogenic groups.

Two MAGs affiliated to Pseudomonadales order (casp40-mb.215 and casp150-mb.169) contain ARGs belonging to categories I, II and III (Supplementary Figure S4). Among 22 ARG containing MAGs affiliated to Gammaproteobacteria, 14 MAGs belonged to Pseudomonadales order with estimated genome sizes in the range of 2.1 to 5.4 Mbp. Among all ARG containing MAGs, the casp40-mb.215 (n = 21 ARGs) and casp150-mb.169 (n = 18 ARGs) affiliated with *Acinetobacter venetianus* had the highest number of ARGs (Supplementary Figure S6). Representatives of genus *Acinetobacter* are commonly found in soil and water^46^. This genus contains *Acinetobacter baumannii* that is a pathogen with known antibiotic resistance complications for infection treatment^47^.

**Figure 4:**
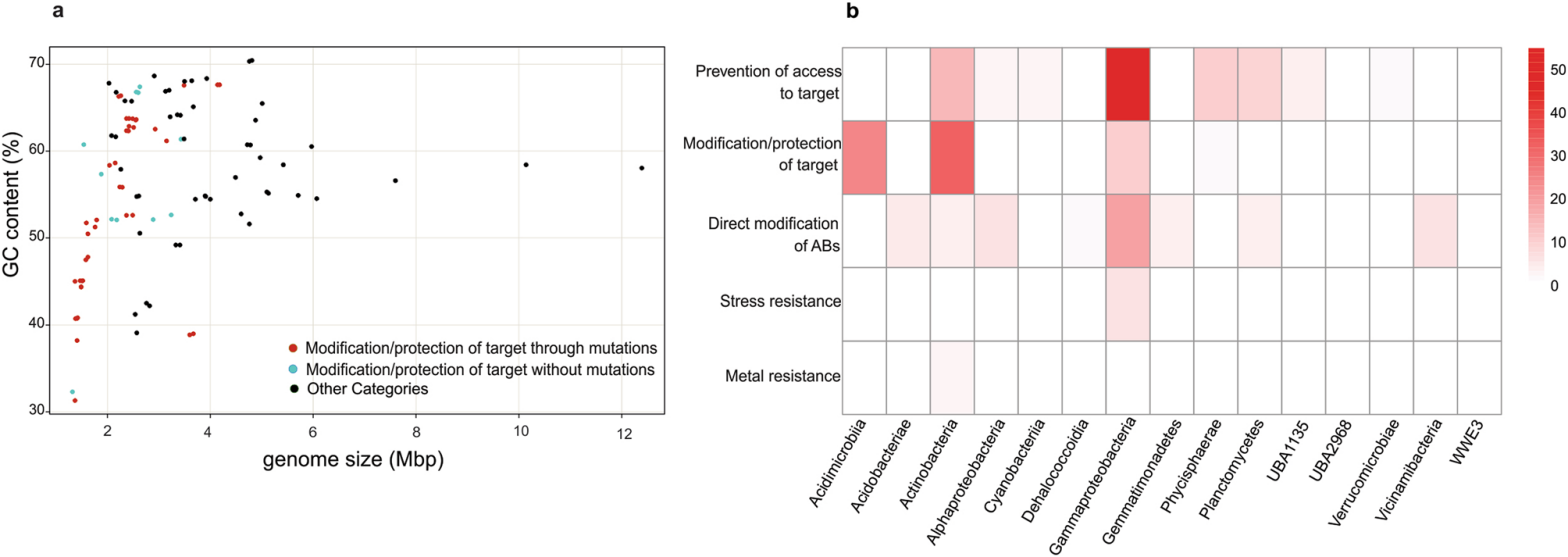
Genome size in ARG containing MAGs. (**a**) Genomic GC content versus estimated genome size for all ARG containing MAGs, red dots indicate genomes that confer category II resistant through mutations, which have lower GC content and estimated genome size. Blue dots indicate genomes that confer category II resistant without mutation. Other categories are shown in Supplementary Figure S3. (**b**) Heat map representation of number of genomes at the taxonomic level of class and ARG categories. Classes of Acidimicrobiia and Actinobacteria have a higher number of ARG category II.

Representatives of the Acidimicrobiia class are ubiquitous aquatic microbes with high relative abundances in the brackish Caspian Sea^36^. These MAGs have the estimated genome size in the range of 1.3 to 2.9 Mbp and their ARGs belong to the category II and are mainly caused by mutations (Fig. 4 and Supplementary Figure S3). In addition to the Acidimicrobiia class, there is a high frequency of antibiotic resistance mechanisms based on target modification and protection detected in the Actinobacteria affiliated MAGs (Fig. 4). For streamlined members of this taxon that are highly abundant in the ecosystem and have adapted to the oligotrophic environments, it could potentially be advantageous to modify the antibiotics target to develop antibiotic resistance so they can avoid the cost of carrying a new gene for developing resistant phenotype. 12 MAGs affiliated to Nanopelagicales order in Actinobacteria class contain 20 ARGs. All these ARGs are in category II and subcategories mutation in rpoB and rpsL genes (12 rpoB gene and 8 rpsL gene). Unlike other Actinobacteria, members of the order Nanopelagicales, family Nanopelagicaceae and AcAMD-5, have a low G + C% content (38% to 47%) in their genome and have streamlined genomes in the range of 1.3 to 1.6 Mbp. Members of this order are present in freshwater and brackish environments such as the Caspian Sea in high abundances making up more than 30% of the microbial community in the surface layer of freshwater ecosystems^48^. According to streamlining theory, these organisms remove unnecessary genes from their genomes, thereby lowering the cellular metabolic costs^49^. In line with this strategy, the use of antibiotic resistance mechanisms based on modification or protection of the target, especially based on mutations in the antibiotic target, seems to be one of the best options to achieve antibiotic resistance in members of such lineages (Supplementary Figure S3). Although this order does not contain a known pathogenic representative, their ubiquitously high abundance in the ecosystem could offer a new perspective on the ecological role of antibiotic resistance genes. The family S36-B12 from order Nanopelagicales that is one of the high G + C% content (about 60%) groups with the estimated genome size in the range of 2–3 Mbp, also developed their resistance through mutations. While we attribute the resistant streamlined genomes to the target modification and protection mechanisms (especially based on mutations), this may be a feature of class Actinobacteria.

Among all ARG containing MAGs, class Acidimicrobiia affiliated MAGs show the highest abundance in three depths of Caspian Sea followed by class Actinobacteria (Supplementary Figure S5). The MAG of casp15-mb.93 and casp15-mb.71 are among the most abundant bacteria with detected ARGs in 40 and 150 m depth metagenomes. These MAGs belong to order Microtrichales and their ARGs were classified in category II having mutations in the rpsL genes. While these MAGs were reconstructed from the 15 m depth metagenomes, they show a higher abundance at the lower strata (Supplementary Figure S5).

Prior culture based studies on the Zarjoub^50^ and Gowharrood^51^ rivers that are entering the Caspian Sea basin report antibiotic resistant coliform bacteria. These studies do not report the antibiotic concentrations of the natural environment but claim that presence of antibiotic resistant bacteria is due to uncontrolled discharge of agricultural and livestock effluents upstream of the river and the entry of municipal and hospital wastewater into these two rivers and later the Caspian Sea. Hence, it is important to understand the accurate resistome profile of this natural ecosystem as a step toward sustaining its ecosystem services.

The Caspian Sea ARG containing MAGs are dominated by representatives of Acidimicrobiia, Gammaproteobacteria and Actinobacteria classes. A recent study on the deep-sea water (more than 1000 m deep) suggest that even deep marine environments could be an environmental reservoir for ARGs mainly carried by representatives of Gammaproteobacteria (70%) and Alphaproteobacteria (20%)^28^. The identified ARGs were classified based on the classes of antibiotics they provide resistance to and most abundant identified ARG types respectively included multidrug, peptide and aminoglycoside^28^. Exploring the diversity and abundance of ARGs in global ocean metagenomes using machine-learning approach (DeepARG tool) showed that ARGs conferring resistance to tetracycline are the most widespread followed by those providing resistance to multidrug and β-lactams. In the contigs containing ARGs, Alphaproteobacteria was identified as the largest taxonomic unit, followed by Gammaproteobacteria^23^. In the Caspian Sea however, similar to the global ocean^23^ most identified ARGs provide resistance to multidrug class followed by β-lactams (Fig. 3). Caspian ARGs conferring resistance to tetracycline were annotated as transporter groups and consequently we classified them into category I and multidrug class. We additionally explored the distribution of ARGs in Caspian viral contigs and six viral contigs identified by virsorter2 contained ARGs however in the follow up manual curations we could not confirm the viral origin of these contigs and removed them from the results.

### Phylogenetic analysis of the Caspian Sea β-lactamases

**Figure 5:**
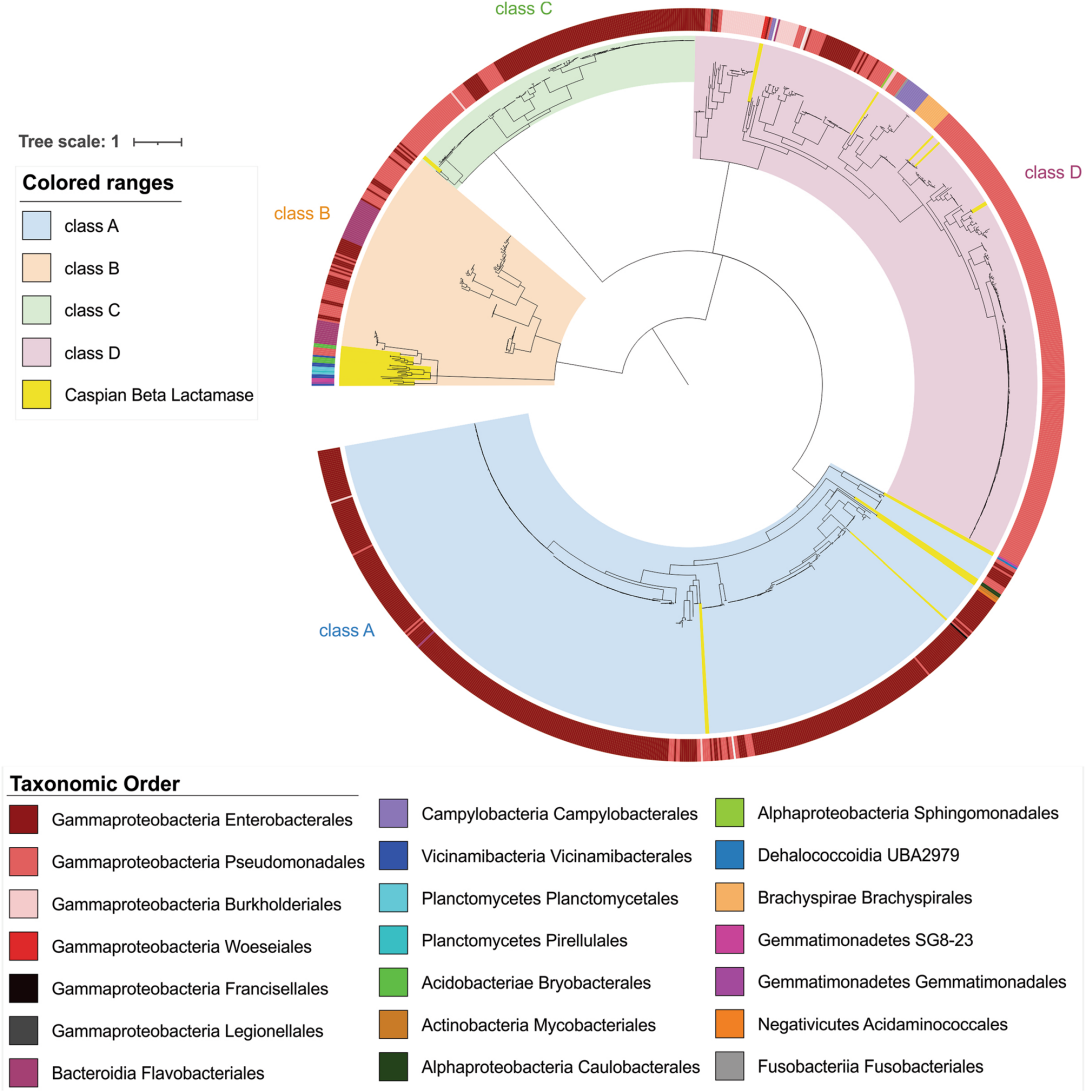
Maximum-likelihood phylogenetic tree of β-lactamases. β-lactamases are classified into four classes based on their amino acid sequences (A to D classes). Phylogenetic tree was constructed by using the maximum likelihood method, and 100 bootstrap replications. Taxonomy of *bla* containing genomes at the order level is annotated on the phylogenetic tree. Caspian β-lactamases are highlight in yellow in the tree.

A total of 40 ARGs classified as β-lactamase genes (*bla*), were detected in the MAGs of the Caspian Sea and their phylogenetic relations were analyzed (reference sequences and tree file are accessible in Supplementary Data File S1). Beta-lactamases are classified into four molecular classes (A to D classes) based on their amino acid sequences. Class A, C, and D enzymes utilize serine for β-lactam hydrolysis and class B are metalloenzymes that require divalent zinc ions for substrate hydrolysis^52^. Among identified Caspian *bla,* 9, 22, 2, and 7 are classified in respectively class A, B, C, and D (Supplementary Table S4). As shown in Fig. 5, most of the Caspian *bla* are metallo-β-lactamases. Metallo-beta-lactamase enzymes pose a particular challenge to drug development due to their structure and diversity^53^. These enzymes escape most of the recently licensed beta-lactamase inhibitors. Acquired metallo-beta-lactamases, which are prevalent in Enterobacterales and *Pseudomonas aeruginosa*, are usually associated with highly drug-resistant phenotypes and are more dangerous^53^. While the *bla* containing reference genes included in this phylogeny (collected from the KEGG database) mostly belong to the Gammaproteobacteria, Caspian *bla* containing genomes represent a higher diverse belonging to six different phyla (in 8 different classes). Some of these *bla* containing MAGs are affiliated to taxa that do not yet have a representative in culture. Natural ecosystems are known to be important reservoirs of β-lactamase gene homologs, however, exchange of β-lactamases between natural environments and human and bovine fecal microbiomes occurs at low frequencies^54^. Additionally, β-lactams can be used as a source of nutrient after β-lactamase cleavage. The β-lactam catabolism pathway has been detected in diverse Proteobacteria isolates from soil that is generating carbon sources for central metabolism^42,55^.

## Conclusions

Antibiotic resistance is a global health challenge and according to One Health approach, attention to environmental antibiotic resistome is critical to combat AMR. Our study shows the distribution of antibiotic resistance genes in the Caspian Sea ecosystem, even though no accurate measurement of antibiotic contamination of the Caspian Sea has been reported so far. Moreover, our findings revealed the mechanism of resistance in streamlined genomes, which is based on target modifications. The increase of antibiotic concentrations in natural ecosystems, as a consequence of human activities, not only influences the Prevalence of antibiotic resistance genes, but also can alter the microbial populations and communities of the Caspian Sea. It can have adverse effects on the carbon and nitrogen cycle balance and hence may cause imbalance in the homeostasis of microbial communities in the Caspian Sea leading to potentially severe consequences for this ecosystem as a whole. However, as bacterial communities are formed by a complex array of evolutionary, ecological and environmental factors, it is difficult to obtain a clear understanding of the evolutionary and ecological consequences of antibiotic resistance in natural environments. The resistome profile and the type of resistance mechanism of the Caspian Sea MAGs provided in this study can be used as a reference database for monitoring the development and spread of antibiotic resistance in the Caspian Sea over time and can also guide future studies. Eventually, Global problems require global solutions and only a concerted and sustained international effort can succeed in dealing with AMR.

## Methods

### Assembly and binning of the Caspian Sea metagenomes

Brackish Caspian Sea metagenome were used for in-silico screening of ARGs. Metagenomic datasets derived from three different depths of the Caspian Sea (15 m, 40 m, and 150 m), were published in 2016 by Mehrshad et al.^36^ and are accessible under the BioProject identifier PRJNA279271. Briefly, a single depth profile was obtained on 1 October 2013 from the southern part of the Caspian Sea. Samples were taken from depths in the ranges of 14 to 25, 39 to 50, and 149 to 160 m using a Rosette Niskin bottle sampler. Physicochemical characteristics of the samples are provided in the original publication^36^. To retrieve the biomass, samples were passed through 0.22 µm filters and these filters containing the biomass were stored on dry ice and transported to the laboratory for DNA extraction. DNA was extracted by a standard phenol–chloroform protocol^56^ and sequenced by use of an Illumina HiSeq 2000 PE101 sequencer. The sequenced metagenomes were quality checked using bbduk.sh script (https://sourceforge.net/projects/bbmap) and assembled using metaSPAdes^57^. Metagenomic reads were mapped against assembled contigs using bbmap.sh script (https://sourceforge.net/projects/bbmap). Contigs ≥ 2 kb were binned based on differential coverage and composition using Metabat2^58^. Quality of the reconstructed MAGs was assessed using CheckM^59^ and bins with completeness ≥ 40% and contamination ≤ 5% were used for further analysis. Taxonomy of these MAGs was assigned using GTDB-tk (v0.3.2) and genome taxonomy database release R89^60^. MAG abundances in different metagenomes of the Caspian sea were calculated using the CoverM tool with transcript per million (TPM) method (https://github.com/wwood/CoverM).

### ARG identification

The ARGs in the Caspian Sea MAGs were determined using the six different pipelines and software (RGI, AMRFinder, ResFinder, sraX, DeepARG, ABRicate equipped with ARG-ANNOT) (Supplementary Table S1). Protein coding sequences of each MAG were predicted using Prodigal^61^. The protein sequences of the reconstructed MAGs were searched for ARGs against the Comprehensive Antibiotic Resistance Database (CARD) using Web portal RGI 5.1.1, CARD 3.1.1 (https://card.mcmaster.ca/analyze/rgi) with default settings^62^.

NCBI AMRFinderPlus v3.9.3 (https://github.com/ncbi/amr/wiki) command line tool and its associated database, The Bacterial Antimicrobial Resistance Reference Gene Database (which contains 4,579 antimicrobial resistance proteins and more than 560 HMMs), were used for screening ARGs. The protein sequences of all reconstructed MAGs were analyzed with parameter "-p"^63^. Additionally, all ARGs present in the MAGs protein sequences were screened using a deep learning approach, DeepARG v1.0.2 command line tool, (https://bitbucket.org/gusphdproj/deeparg-ss/src/master/) with DeepARG-DB database (–model LS–type nucl–arg-alignment-identity 60)^64^.

The nucleotide sequences of the reconstructed MAGs were searched for ARGs using ResFinder 4.1 command line tool (https://bitbucket.org/genomicepidemiology/resfinder/src/master/) and its associated database, ResFinder database with parameters "-ifa -acq -l 0.6 -t 0.8"^65^. They were also searched using ARGminer v1.1.1 database^66^ and BacMet v2.0 database^67^ using sraX v1.5 command line tool (https://github.com/lgpdevtools/srax) with parameters "-db ext –s blastx"^68^. These sequences were also searched against ARG-ANNOT v4 database^69^ using ABRicate v0.8 command line tool^70^.

Results of these methods presented candidate ARGs in our MAG set. Functions of the ARG candidates were further verified using five different annotation tools (default settings); Batch web conserved domain search (CD-Search) in NCBI https://www.ncbi.nlm.nih.gov/Structure/bwrpsb/bwrpsb.cgi^71^, web-based Hmmer v2.41.1 (phmmer) https://www.ebi.ac.uk/Tools/hmmer/search/phmmer^72^, hmmscan against Pfam v34.0 database http://pfam.xfam.org/search#tabview=tab1^73^, GhostKOALA v2.2https://www.kegg.jp/ghostkoala/^74^, and eggNOG-mapper v2 http://eggnog-mapper.embl.de/^75^. All functional annotation results were compiled and results were compared to obtain a consensus assignment. Then, ARGs were manually curated into 5 antibiotic resistance categories and 21 subcategories based on their functional annotations. The overall workflow of this study is shown in Supplementary Figure S8.

### Gene alignment

To confirm resistance due to mutation events in the candidate Caspian ARGs, multiple amino acid sequence alignment was carried out Using Clustal-W (default parameters)^76^ embedded in MEGA-X software ^77^. For each type of the ARGs, reference gene with specific mutations was downloaded from CARD database (Supplementary Table S2 shows the detail of mutations involved in antibiotic resistance).

### Beta-lactamase phylogeny

To understand the evolutionary relationship of the recovered β-lactamase enzymes, firstly, 1141 reference protein sequences (beta-Lactamase gene variants) were downloaded from KEGG database, https://www.genome.jp/kegg/annotation/br01553.html and combined with β-lactamases recovered from Caspian MAGs (40 protein sequences). Then, all β-lactamase sequences were subjected to multiple sequence alignment using Clustal-W embedded in MEGA-X (Molecular Evolutionary Genetics Analysis) software^77^. Phylogenetic tree was constructed using the maximum likelihood method, JTT matrix-based model, and 100 bootstrap replications in MEGA-X software. The bootstrap consensus tree inferred from 100 replicates is taken to represent the evolutionary history. This analysis involved 1181 amino acid sequences in total. Taxonomic assignment of MAGs was extended to the of β-lactamases and iTOL v6.3.1 was used to annotate and visualize the final phylogenetic tree^78^.

### Identification of viral contigs

Viral contigs were identified in contigs longer than 1 kb using VirSorter2 tool at the score threshold of 0.8^79^. These contigs were further checked manually to ensure the viral origin.

## Supplementary Information

Supplementary Information 1.

Supplementary Information 2.

Supplementary Information 3.

Supplementary Information 4.

## Data Availability

The Caspian Sea metagenomes used for this study have been deposited to GenBank by Mehrshad et al.^36^ and are accessible via the bioproject PRJNA279271. Genomes containing ARGs were also deposited to GenBank and are accessible under the accession number Bioproject PRJNA279271. All alignments used for manual evaluation of mutations, detailed stats of detected ARGs, and their sequences are accompanying this manuscript as Supplementary data S2.
