# Supplementary material for: Genome-resolved insight into the reservoir of antibiotic resistance genes in aquatic microbial community
Source: Sci Rep. 2022 Dec 6;12:21047. doi: 10.1038/s41598-022-25026-3 (PMC9726936; doi:10.1038/s41598-022-25026-3)
Supplement: Supplementary file 1 — Supplementary Information 1. [file 41598_2022_25026_MOESM1_ESM.pdf]

Title: Genome-resolved insight into the reservoir of antibiotic resistance genes in aquatic microbial community

Authors: Zahra Goodarzi[1], Sedigheh Asad[1], Maliheh Mehrshad[2]

[1] Department of Biotechnology, College of Science, University of Tehran, Tehran, Iran

[2] Department of Aquatic Sciences and Assessment, Swedish University of Agricultural Sciences (SLU), Box 7050, SE75007 Uppsala, Sweden

Supplementary Table S1. Summary of ARG identification tools and databases used in this study.

Tool	Database	Approach	Input
**RGI**	CARD	BLASTp	Protein sequence
**AMRFinder (NCBI)**	The Bacterial Antimicrobial Resistance Reference Gene Database	BLASTp & HMM scan	Protein sequence
**DeepARG**	DeepARG-DB	deep-learning	Protein sequence
**ResFinder**	ResFinder	BLASTn	Nucleotide sequence
**sraX**	CARD & ARGminer & BacMet	BLASTx	Nucleotide sequence
**ABRicate**	ARG-ANNOT	BLASTn	Nucleotide sequence

Supplementary Table S2. Reference genes and the mutation position for each of ARGs that confirm resistance due to mutation events.

Gene symbol	Gene name	# genes	Reference gene	Mutation position	Ref.
**rpoB**	DNA-directed RNA polymerase subunit beta	20	NP_215181<br>CCP43410.1	L511R<br>D516G<br>H526T, E250G	https://card.mcmaster.ca/ontology/39867
**rpsL**	30S ribosomal protein S12	43	WP_003910993	K43R<br>K88R	https://card.mcmaster.ca/ontology/39979
**rpsA**	30S ribosomal protein S1	3	WP_016810233<br>CCP44394	D123A<br>Δ438A<br>A412V	https://card.mcmaster.ca/ontology/42776

**gyrA**	DNA gyrase subunit A (Topoisomerase)	7	WP_023644204<br>WP_000116442	A90V, D94G<br>G79C, S81L	https://card.mcmaster.ca/ontology/39879, https://card.mcmaster.ca/ontology/40507
**parC**	Topoisomerase IV subunit A	2	WP_000202252	S84L, D105E	https://card.mcmaster.ca/ontology/40508
**LpxC**	UDP-3-O-acyl-N-acetylglucosamine deacetylase	4	AJF83452	P30L	https://card.mcmaster.ca/ontology/40184
**murA**	UDP-N-acetylglucosamine enolpyruvyl transferase	3	CCE36834	C117D	https://card.mcmaster.ca/ontology/40467

Supplementary Table S3. Taxonomy of the Caspian Sea antibiotic resistant MAGs. The percent of completeness and contamination of these MAGs with their estimated genome size, GC content and taxonomic assignments are listed.

Genome id	Estimated Genome size (Mbp)	GC content %	Completeness %	Contamination %	Taxonomy
**casp150-mb.136**	5.97	60.52	92.52	3.42	p__Acidobacteriota;c__Acidobacteriae;o__Bryobacterales; f__UBA6623;g__;s__
**casp150-mb.154**	4.72	60.73	95.67	1.83	p__Acidobacteriota;c__Acidobacteriae;o__Bryobacterales; f__UBA6623;g__;s__
**casp40-mb.95**	4.78	60.70	95.25	3.89	p__Acidobacteriota;c__Acidobacteriae;o__Bryobacterales; f__UBA6623;g__;s__
**casp150-mb.205**	3.67	65.10	93.68	4.27	p__Acidobacteriota;c__Vicinamibacteria;o__Vicinamibacterales; f__UBA2999;g__;s__
**casp150-mb.299**	5.01	65.49	90.6	3.42	p__Acidobacteriota;c__Vicinamibacteria;o__Vicinamibacterales; f__UBA8438;g__UBA8438;s__
**casp15-mb.71**	1.76	51.25	87.09	2.56	p__Actinobacteriota;c__Acidimicrobiia;o__Microtrichales; f__Ilumatobacteraceae;g__BACL27;s__
**casp15-mb.93**	1.59	51.73	90.62	3.68	p__Actinobacteriota;c__Acidimicrobiia;o__Microtrichales; f__Ilumatobacteraceae;g__BACL27;s__
**casp150-mb.152**	1.62	47.79	89.32	0.85	p__Actinobacteriota;c__Acidimicrobiia;o__Microtrichales; f__Ilumatobacteraceae;g__BACL27;s__
**casp150-mb.247**	1.62	50.47	78.66	3.42	p__Actinobacteriota;c__Acidimicrobiia;o__Microtrichales; f__Ilumatobacteraceae;g__BACL27;s__
**casp15-mb.34**	2.42	63.76	93.16	1.28	p__Actinobacteriota;c__Acidimicrobiia;o__Microtrichales; f__Ilumatobacteraceae;g__Casp-actino5;s__
**casp15-mb.84**	2.49	63.73	98.29	3.85	p__Actinobacteriota;c__Acidimicrobiia;o__Microtrichales; f__Ilumatobacteraceae;g__Casp-actino5;s__
**casp150-mb.11**	2.55	63.57	97.44	3.94	p__Actinobacteriota;c__Acidimicrobiia;o__Microtrichales; f__Ilumatobacteraceae;g__Casp-actino5;s__
**casp150-mb.170**	2.37	63.76	93.16	2.14	p__Actinobacteriota;c__Acidimicrobiia;o__Microtrichales; f__Ilumatobacteraceae;g__Casp-actino5;s__
**casp150-mb.321**	2.22	66.30	91.45	3.85	p__Actinobacteriota;c__Acidimicrobiia;o__Microtrichales; f__Ilumatobacteraceae;g__Casp-actino5;s__
**casp40-mb.212**	2.56	63.64	96.58	1.28	p__Actinobacteriota;c__Acidimicrobiia;o__Microtrichales; f__Ilumatobacteraceae;g__Casp-actino5;s__

**casp40-mb.94**	2.26	66.38	82.34	3.85	p__Actinobacteriota;c__Acidimicrobiia;o__Microtrichales; f__Ilumatobacteraceae;g__Casp-actino5;s__
**casp15-mb.191**	2.29	55.83	94.02	1.28	p__Actinobacteriota;c__Acidimicrobiia;o__Microtrichales; f__Ilumatobacteraceae;g__Casp-actino5;s__Casp-actino5 sp001510385
**casp40-mb.42**	2.24	55.86	94.02	1.28	p__Actinobacteriota;c__Acidimicrobiia;o__Microtrichales; f__Ilumatobacteraceae;g__Casp-actino5;s__Casp-actino5 sp001510385
**casp15-mb.86**	2.41	62.32	83.59	0.85	p__Actinobacteriota;c__Acidimicrobiia;o__Microtrichales; f__Ilumatobacteraceae;g__Casp-actino8;s__
**casp150-mb.228**	2.37	62.35	80.6	0.85	p__Actinobacteriota;c__Acidimicrobiia;o__Microtrichales; f__Ilumatobacteraceae;g__Casp-actino8;s__
**casp40-mb.149**	2.39	62.35	83.16	0.85	p__Actinobacteriota;c__Acidimicrobiia;o__Microtrichales; f__Ilumatobacteraceae;g__Casp-actino8;s__
**casp150-mb.258**	2.37	52.60	97.61	3.07	p__Actinobacteriota;c__Acidimicrobiia;o__Microtrichales; f__Ilumatobacteraceae;g__Casp-actino8;s__Casp-actino8 sp001510335
**casp40-mb.115**	2.49	52.61	96.3	2.14	p__Actinobacteriota;c__Acidimicrobiia;o__Microtrichales; f__Ilumatobacteraceae;g__Casp-actino8;s__Casp-actino8 sp001510335
**casp150-mb.41**	2.51	62.73	94.02	2.99	p__Actinobacteriota;c__Acidimicrobiia;o__Microtrichales; f__Ilumatobacteraceae;g__Casp-actino8;s__Casp-actino8 sp001510455
**casp40-mb.285**	2.42	62.86	94.02	1.71	p__Actinobacteriota;c__Acidimicrobiia;o__Microtrichales; f__Ilumatobacteraceae;g__Casp-actino8;s__Casp-actino8 sp001510455
**casp40-mb.234**	2.93	62.53	93.16	2.56	p__Actinobacteriota;c__Acidimicrobiia;o__Microtrichales; f__Ilumatobacteraceae;g__Ilumatobacter;s__
**casp15-mb.62**	1.79	52.06	88.08	0.95	p__Actinobacteriota;c__Acidimicrobiia;o__Microtrichales; f__Ilumatobacteraceae;g__UBA3006;s__UBA3006 sp001438985
**casp150-mb.29**	1.37	31.31	75.21	2.14	p__Actinobacteriota;c__Acidimicrobiia;o__TMED189; f__TMED189;g__TMED189;s__
**casp150-mb.86**	2.04	58.36	95.73	3.56	p__Actinobacteriota;c__Acidimicrobiia;o__UBA5794; f__UBA5794;g__UBA5794;s__
**casp15-mb.16**	3.49	67.58	70.15	0.05	p__Actinobacteriota;c__Actinobacteria;o__Mycobacteriales; f__Mycobacteriaceae;g__Mycolicibacterium;s__
**casp150-mb.119**	4.18	67.64	96.53	1.61	p__Actinobacteriota;c__Actinobacteria;o__Mycobacteriales; f__Mycobacteriaceae;g__Mycolicibacterium;s__Mycolicibacterium sp001510415
**casp40-mb.75**	4.14	67.64	97.15	0.94	p__Actinobacteriota;c__Actinobacteria;o__Mycobacteriales; f__Mycobacteriaceae;g__Mycolicibacterium;s__Mycolicibacterium sp001510415
**casp40-mb.59**	1.37	44.99	67.91	1.94	p__Actinobacteriota;c__Actinobacteria;o__Nanopelagicales; f__AcAMD-5;g__AcAMD-5;s__

**casp150-mb.328**	1.41	38.22	64.81	0.59	p__Actinobacteriota;c__Actinobacteria;o__Nanopelagicales; f__AcAMD-5;g__ATZT02;s__
**casp15-mb.194**	1.51	45.04	89.92	1.23	p__Actinobacteriota;c__Actinobacteria;o__Nanopelagicales; f__Nanopelagicaceae;g__AAA044-D11;s__AAA044-D11 sp002340925
**casp150-mb.71**	1.47	45.06	86.95	0.66	p__Actinobacteriota;c__Actinobacteria;o__Nanopelagicales; f__Nanopelagicaceae;g__AAA044-D11;s__AAA044-D11 sp002340925
**casp40-mb.60**	1.52	45.07	90.19	1.89	p__Actinobacteriota;c__Actinobacteria;o__Nanopelagicales; f__Nanopelagicaceae;g__AAA044-D11;s__AAA044-D11 sp002340925
**casp40-mb.83**	1.49	44.37	83.65	0.86	p__Actinobacteriota;c__Actinobacteria;o__Nanopelagicales; f__Nanopelagicaceae;g__MAG-120802;s__
**casp15-mb.46**	1.42	40.84	71.59	0.79	p__Actinobacteriota;c__Actinobacteria;o__Nanopelagicales; f__Nanopelagicaceae;g__Nanopelagicus;s__Nanopelagicus sp001437855
**casp150-mb.270**	1.38	40.74	66.2	2.81	p__Actinobacteriota;c__Actinobacteria;o__Nanopelagicales; f__Nanopelagicaceae;g__Nanopelagicus;s__Nanopelagicus sp001437855
**casp40-mb.272**	1.41	40.76	74.4	0.7	p__Actinobacteriota;c__Actinobacteria;o__Nanopelagicales; f__Nanopelagicaceae;g__Nanopelagicus;s__Nanopelagicus sp001437855
**casp150-mb.195**	1.58	47.47	88.52	0.7	p__Actinobacteriota;c__Actinobacteria;o__Nanopelagicales; f__Nanopelagicaceae;g__Planktophila;s__
**casp40-mb.119**	2.15	58.63	94.61	0.81	p__Actinobacteriota;c__Actinobacteria;o__Nanopelagicales; f__S36-B12;g__GCA-2737125;s__
**casp15-mb.155**	3.15	61.17	76.47	2.34	p__Actinobacteriota;c__Actinobacteria;o__Nanopelagicales; f__S36-B12;g__S36-B12;s__
**casp150-mb.221**	2.26	57.90	82.84	2.42	p__Chloroflexota;c__Dehalococcoidia;o__SAR202; f__;g__;s__
**casp150-mb.66**	2.03	67.82	51.91	4.13	p__Chloroflexota;c__Dehalococcoidia;o__UBA2979; f__UBA2979;g__;s__
**casp15-mb.81**	5.10	55.29	63.68	3.12	p__Cyanobacteria;c__Cyanobacteriia;o__Phormidesmiales; f__Phormidesmiaceae;g__;s__
**casp40-mb.69**	5.13	55.14	87.27	1.63	p__Cyanobacteria;c__Cyanobacteriia;o__Phormidesmiales; f__Phormidesmiaceae;g__;s__
**casp40-mb.232**	4.97	59.24	57.97	0.72	p__Cyanobacteria;c__Cyanobacteriia;o__Phormidesmiales; f__Phormidesmiaceae;g__PCC-6406;s__
**casp150-mb.31**	3.22	63.95	97.8	2.2	p__Gemmatimonadota;c__Gemmatimonadetes;o__Gemmatimonadales; f__Gemmatimonadaceae;g__Fen-1231;s__
**casp150-mb.298**	3.42	64.13	97.8	4.4	p__Gemmatimonadota;c__Gemmatimonadetes;o__SG8-23; f__UBA6960;g__;s__
**casp40-mb.311**	3.36	64.19	98.9	4.95	p__Gemmatimonadota;c__Gemmatimonadetes;o__SG8-23; f__UBA6960;g__;s__
**casp40-mb.18**	5.71	54.89	79.83	1.2	p__Latescibacterota;c__UBA2968;o__UBA2968; f__GCA-2709665;g__;s__

**casp150-mb.130**	1.32	32.31	55.12	0.99	p__Patescibacteria;c__WWE3;o__; f__;g__;s__
**casp15-mb.10**	2.91	68.66	50.24	3.98	p__Planctomycetota;c__Phycisphaerae;o__Phycisphaerales; f__SM1A02;g__;s__
**casp150-mb.166**	2.63	50.54	96.15	0	p__Planctomycetota;c__Phycisphaerae;o__Phycisphaerales; f__SM1A02;g__;s__
**casp150-mb.294**	2.47	65.75	88.35	1.14	p__Planctomycetota;c__Phycisphaerae;o__Phycisphaerales; f__SM1A02;g__;s__
**casp150-mb.65**	1.88	57.34	95.45	0	p__Planctomycetota;c__Phycisphaerae;o__Phycisphaerales; f__SM1A02;g__;s__
**casp40-mb.123**	2.34	65.79	96.59	1.14	p__Planctomycetota;c__Phycisphaerae;o__Phycisphaerales; f__SM1A02;g__;s__
**casp150-mb.61**	3.49	61.40	86.53	1.24	p__Planctomycetota;c__Phycisphaerae;o__Phycisphaerales; f__SM1A02;g__UBA12014;s__
**casp40-mb.35**	3.43	61.37	93.62	2.01	p__Planctomycetota;c__Phycisphaerae;o__Phycisphaerales; f__SM1A02;g__UBA12014;s__
**casp150-mb.46**	10.14	58.42	96.48	3.45	p__Planctomycetota;c__Planctomycetes;o__Pirellulales; f__Pirellulaceae;g__GCA-2726245;s__
**casp150-mb.201**	3.50	68.03	90.74	1.72	p__Planctomycetota;c__Planctomycetes;o__Pirellulales; f__UBA1268;g__;s__
**casp150-mb.45**	4.88	63.56	70.35	2.4	p__Planctomycetota;c__Planctomycetes;o__Pirellulales; f__UBA1268;g__;s__
**casp40-mb.39**	3.64	68.10	97.63	1.15	p__Planctomycetota;c__Planctomycetes;o__Pirellulales; f__UBA1268;g__;s__
**casp150-mb.69**	12.39	58.04	62.5	1.79	p__Planctomycetota;c__Planctomycetes;o__Planctomycetales; f__Planctomycetaceae;g__;s__
**casp40-mb.109**	6.07	54.52	97.69	1.19	p__Planctomycetota;c__Planctomycetes;o__Planctomycetales; f__Planctomycetaceae;g__;s__
**casp40-mb.51**	7.60	56.59	64.69	1.25	p__Planctomycetota;c__Planctomycetes;o__Planctomycetales; f__Planctomycetaceae;g__;s__
**casp150-mb.290**	2.57	54.76	95.16	1.08	p__Planctomycetota;c__UBA1135;o__UBA1135; f__UBA1135;g__GCA-2746235;s__
**casp40-mb.240**	2.61	54.82	94.09	1.08	p__Planctomycetota;c__UBA1135;o__UBA1135; f__UBA1135;g__GCA-2746235;s__
**casp15-mb.161**	4.81	70.44	95.45	1.14	p__Planctomycetota;c__UBA1135;o__UBA2386; f__UBA2386;g__;s__
**casp40-mb.29**	4.76	70.34	89.2	0	p__Planctomycetota;c__UBA1135;o__UBA2386; f__UBA2386;g__;s__
**casp150-mb.133**	3.20	67.01	71.35	3.01	p__Proteobacteria;c__Alphaproteobacteria;o__Caulobacterales; f__Caulobacteraceae;g__Brevundimonas;s__

**casp40-mb.174**	3.13	66.89	85.68	1.1	p__Proteobacteria;c__Alphaproteobacteria;o__Caulobacterales; f__Caulobacteraceae;g__Brevundimonas;s__
**casp40-mb.87**	4.49	56.96	71.02	3.75	p__Proteobacteria;c__Alphaproteobacteria;o__Rhodospirillales_A; f__;g__;s__
**casp40-mb.99**	3.93	68.36	97.85	1.08	p__Proteobacteria;c__Alphaproteobacteria;o__SP197; f__SP197;g__;s__
**casp40-mb.200**	2.54	41.23	43.86	0.43	p__Proteobacteria;c__Alphaproteobacteria;o__Sphingomonadales; f__Emcibacteraceae;g__UBA4441;s__
**casp150-mb.269**	2.17	66.77	75.46	1.2	p__Proteobacteria;c__Alphaproteobacteria;o__UBA2966; f__;g__;s__
**casp150-mb.97**	2.60	66.74	98.1	1.92	p__Proteobacteria;c__Gammaproteobacteria;o__Burkholderiales; f__SG8-39;g__RBG-16-66-20;s__
**casp40-mb.55**	2.56	66.81	80.6	4.72	p__Proteobacteria;c__Gammaproteobacteria;o__Burkholderiales; f__SG8-39;g__RBG-16-66-20;s__
**casp150-mb.292**	2.63	67.41	88.63	1.53	p__Proteobacteria;c__Gammaproteobacteria;o__Burkholderiales; f__SG8-39;g__SG8-39;s__
**casp15-mb.175**	2.57	39.10	91.2	0.29	p__Proteobacteria;c__Gammaproteobacteria;o__Francisellales; f__Francisellaceae;g__Caedibacter;s__
**casp40-mb.114**	4.60	52.76	94.8	4.28	p__Proteobacteria;c__Gammaproteobacteria;o__Ga0077536; f__Ga0077536;g__UBA11873;s__
**casp150-mb.297**	1.54	60.75	92.49	1.17	p__Proteobacteria;c__Gammaproteobacteria;o__Ga0077554; f__Ga007554;g__;s__
**casp40-mb.214**	2.18	52.07	81	0.66	p__Proteobacteria;c__Gammaproteobacteria;o__Pseudomonadales; f__Halieaceae;g__IMCC3088;s__
**casp15-mb.124**	2.08	52.15	71.43	1.49	p__Proteobacteria;c__Gammaproteobacteria;o__Pseudomonadales; f__Halieaceae;g__IMCC3088;s__IMCC3088 sp003520285
**casp15-mb.181**	3.24	52.64	79.8	3.33	p__Proteobacteria;c__Gammaproteobacteria;o__Pseudomonadales; f__Halieaceae;g__Luminiphilus;s__
**casp40-mb.140**	5.42	58.42	87.98	4.69	p__Proteobacteria;c__Gammaproteobacteria;o__Pseudomonadales; f__HTCC2089;g__;s__
**casp150-mb.241**	2.82	42.21	75.66	1.4	p__Proteobacteria;c__Gammaproteobacteria;o__Pseudomonadales; f__Moraxellaceae;g__Acinetobacter;s__Acinetobacter johnsonii
**casp40-mb.142**	2.76	42.52	59.01	0.83	p__Proteobacteria;c__Gammaproteobacteria;o__Pseudomonadales; f__Moraxellaceae;g__Acinetobacter;s__Acinetobacter johnsonii
**casp150-mb.169**	3.60	38.87	98.18	1.73	p__Proteobacteria;c__Gammaproteobacteria;o__Pseudomonadales; f__Moraxellaceae;g__Acinetobacter;s__Acinetobacter venetianus
**casp40-mb.215**	3.67	38.99	98.53	0.79	p__Proteobacteria;c__Gammaproteobacteria;o__Pseudomonadales; f__Moraxellaceae;g__Acinetobacter;s__Acinetobacter venetianus
**casp15-mb.5**	2.89	52.11	94.81	2.16	p__Proteobacteria;c__Gammaproteobacteria;o__Pseudomonadales; f__Pseudohongiellaceae;g__OM182;s__

**casp15-mb.167**	3.91	54.77	95	1.17	p__Proteobacteria;c__Gammaproteobacteria;o__Pseudomonadales; f__Pseudohongiellaceae;g__UBA9145;s__
**casp150-mb.100**	4.76	51.60	94.63	1.98	p__Proteobacteria;c__Gammaproteobacteria;o__Pseudomonadales; f__Pseudohongiellaceae;g__UBA9145;s__
**casp150-mb.148**	3.33	49.18	94.07	1.79	p__Proteobacteria;c__Gammaproteobacteria;o__Pseudomonadales; f__Pseudohongiellaceae;g__UBA9145;s__
**casp40-mb.220**	3.90	54.83	90.06	1.94	p__Proteobacteria;c__Gammaproteobacteria;o__Pseudomonadales; f__Pseudohongiellaceae;g__UBA9145;s__
**casp40-mb.61**	3.41	49.18	94.07	1.23	p__Proteobacteria;c__Gammaproteobacteria;o__Pseudomonadales; f__Pseudohongiellaceae;g__UBA9145;s__
**casp150-mb.9**	4.00	54.45	89.06	2.81	p__Proteobacteria;c__Gammaproteobacteria;o__Woeseiales; f__Woeseiaceae;g__SZUA-117;s__
**casp40-mb.225**	3.71	54.45	88.41	2.51	p__Proteobacteria;c__Gammaproteobacteria;o__Woeseiales; f__Woeseiaceae;g__SZUA-117;s__
**casp150-mb.222**	2.16	61.65	97.97	0.68	p__Verrucomicrobiota;c__Verrucomicrobiae;o__Chthoniobacterales; f__;g__;s__
**casp40-mb.44**	2.08	61.78	97.8	1.01	p__Verrucomicrobiota;c__Verrucomicrobiae;o__Chthoniobacterales; f__;g__;s__

Supplementary Table S4. List of β-lactamase containing genomes in the Caspian Sea MAGs and their taxonomic affiliation. There are 40 β-lactamase genes detected in 30 Caspian Sea MAGs.

Genome id	Gene id	β-lactamase class	Class	Order
**casp150-mb.136**	EEKHDION 03010	class B	Acidobacteriae	Bryobacterales
**casp150-mb.154**	EOCCHKBK 01016	class B	Acidobacteriae	Bryobacterales
	EOCCHKBK 03411	class B		
**casp40-mb.95**	OICPLIJP 01112	class B	Acidobacteriae	Bryobacterales
	OICPLIJP 03168	class B		
**casp150-mb.119**	KIBDMIMN 02996	class A	Actinobacteria	Mycobacteriales
**casp40-mb.75**	BGPOMPEL 02839	class A	Actinobacteria	Mycobacteriales
**casp150-mb.133**	IBHFGKKE 00487	class A	Alphaproteobacteria	Caulobacterales
**casp40-mb.174**	LEECDGGH 01667	class A	Alphaproteobacteria	Caulobacterales
**casp40-mb.200**	IKHHLIND 00205	class D	Alphaproteobacteria	Sphingomonadales
**casp150-mb.66**	IFOMPFBH 00010	class A	Dehalococcoidia	UBA2979
**casp15-mb.175**	EBJODPFO 00650	class A	Gammaproteobacteria	Francisellales
**casp15-mb.167**	ADILNDBO 00237	class B	Gammaproteobacteria	Pseudomonadales
**casp150-mb.100**	POANLGLH 03091	class B	Gammaproteobacteria	Pseudomonadales
	POANLGLH 03261	class B		
**casp150-mb.148**	JKEJJGBH 00119	class A	Gammaproteobacteria	Pseudomonadales
**casp150-mb.169**	HNBCKAMJ 00045	class C	Gammaproteobacteria	Pseudomonadales
	HNBCKAMJ 00307	class D		
**casp150-mb.241**	HMCNMLOA 00746	class D	Gammaproteobacteria	Pseudomonadales
**casp40-mb.142**	FCMKGLIF 00920	class D	Gammaproteobacteria	Pseudomonadales
**casp40-mb.215**	IAHFBJBJ 00371	class D	Gammaproteobacteria	Pseudomonadales

	IAHFBJBJ 00481	class C		
**casp40-mb.220**	GPCFOBLN 01961	class B	Gammaproteobacteria	Pseudomonadales
**casp40-mb.61**	GHFBJIFD 00366	class A	Gammaproteobacteria	Pseudomonadales
**casp150-mb.9**	MFLPOMFE 01486	class D	Gammaproteobacteria	Woeseiales
**casp40-mb.225**	CLDJHIBN 02085	class D	Gammaproteobacteria	Woeseiales
**casp150-mb.31**	CFBKJOIE 01731	class B	Gemmatimonadetes	Gemmatimonadales
**casp150-mb.298**	NPIGMJFH 02028	class B	Gemmatimonadetes	SG8-23
**casp40-mb.311**	OAPKMILO 00770	class A	Gemmatimonadetes	SG8-23
	OAPKMILO 01670	class B		
**casp150-mb.46**	AIEJOLAH 07624	class B	Planctomycetes	Pirellulales
**casp150-mb.69**	BIDOACPM 03738	class B	Planctomycetes	Planctomycetales
**casp40-mb.109**	PCMHCDHE 03871	class B	Planctomycetes	Planctomycetales
**casp40-mb.51**	CDIDJOII 03913	class B	Planctomycetes	Planctomycetales
**casp150-mb.205**	KEACKLOE 00888	class B	Vicinamibacteria	Vicinamibacterales
	KEACKLOE 02795	class B		
**casp150-mb.299**	BNEENLPF 00151	class B	Vicinamibacteria	Vicinamibacterales
	BNEENLPF 02856	class B		
	BNEENLPF 03106	class B		
	BNEENLPF 03234	class B		
