# Supplementary material for: Genome-resolved insight into the reservoir of antibiotic resistance genes in aquatic microbial community
Source: Sci Rep. 2022 Dec 6;12:21047. doi: 10.1038/s41598-022-25026-3 (PMC9726936; doi:10.1038/s41598-022-25026-3)
Supplement: Supplementary file 2 — Supplementary Information 2. [file 41598_2022_25026_MOESM2_ESM.pdf]

Title: Genome-resolved insight into the reservoir of antibiotic resistance genes in aquatic microbial community

Authors: Zahra Goodarzi[1], Sedigheh Asad[1], Maliheh Mehrshad[2]

[1] Department of Biotechnology, College of Science, University of Tehran, Tehran, Iran

[2] Department of Aquatic Sciences and Assessment, Swedish University of Agricultural Sciences (SLU), Box 7050, SE75007 Uppsala, Sweden

Supplementary figures

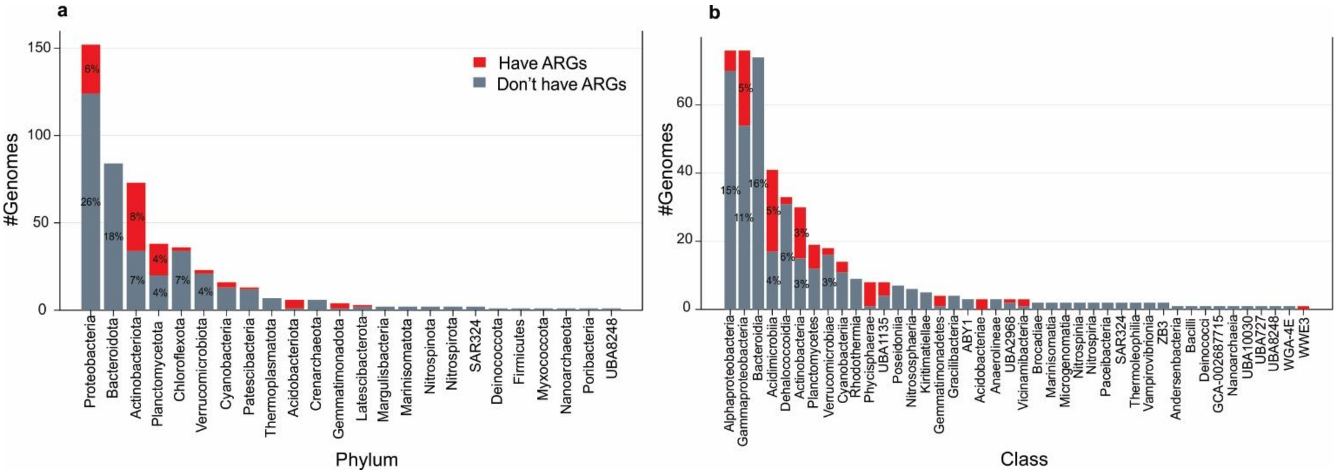

Figure S1. Overall taxonomic distribution of all reconstructed MAGs from the Caspian Sea metagenomes at phylum (**a**) and class (**b**) level. The red areas indicate the number of ARG containing MAGs in each taxa.

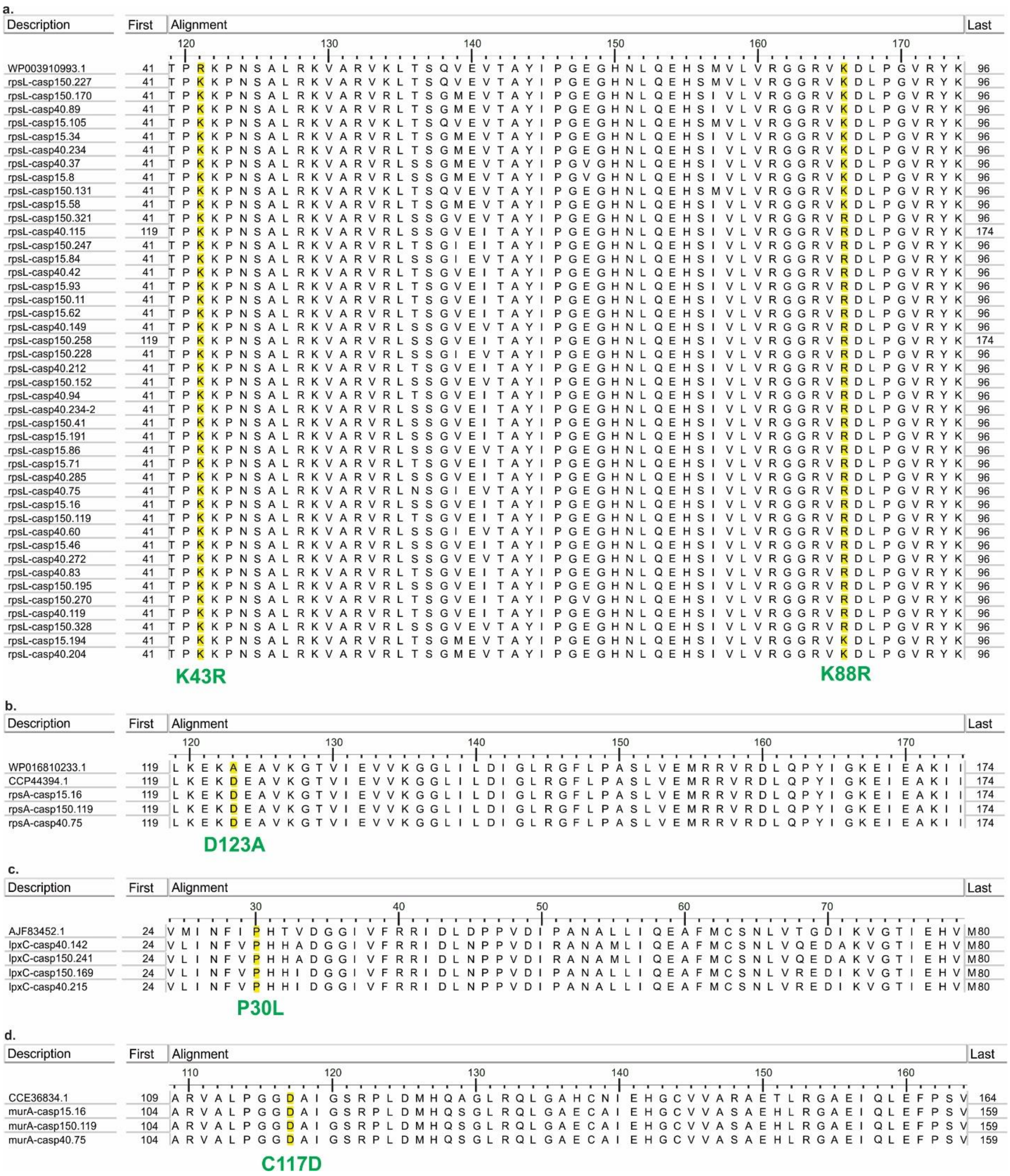
a.
Description
First
Alignment
Last
K43R
K88R
b.
D123A
c.
P30L
d.
C117D

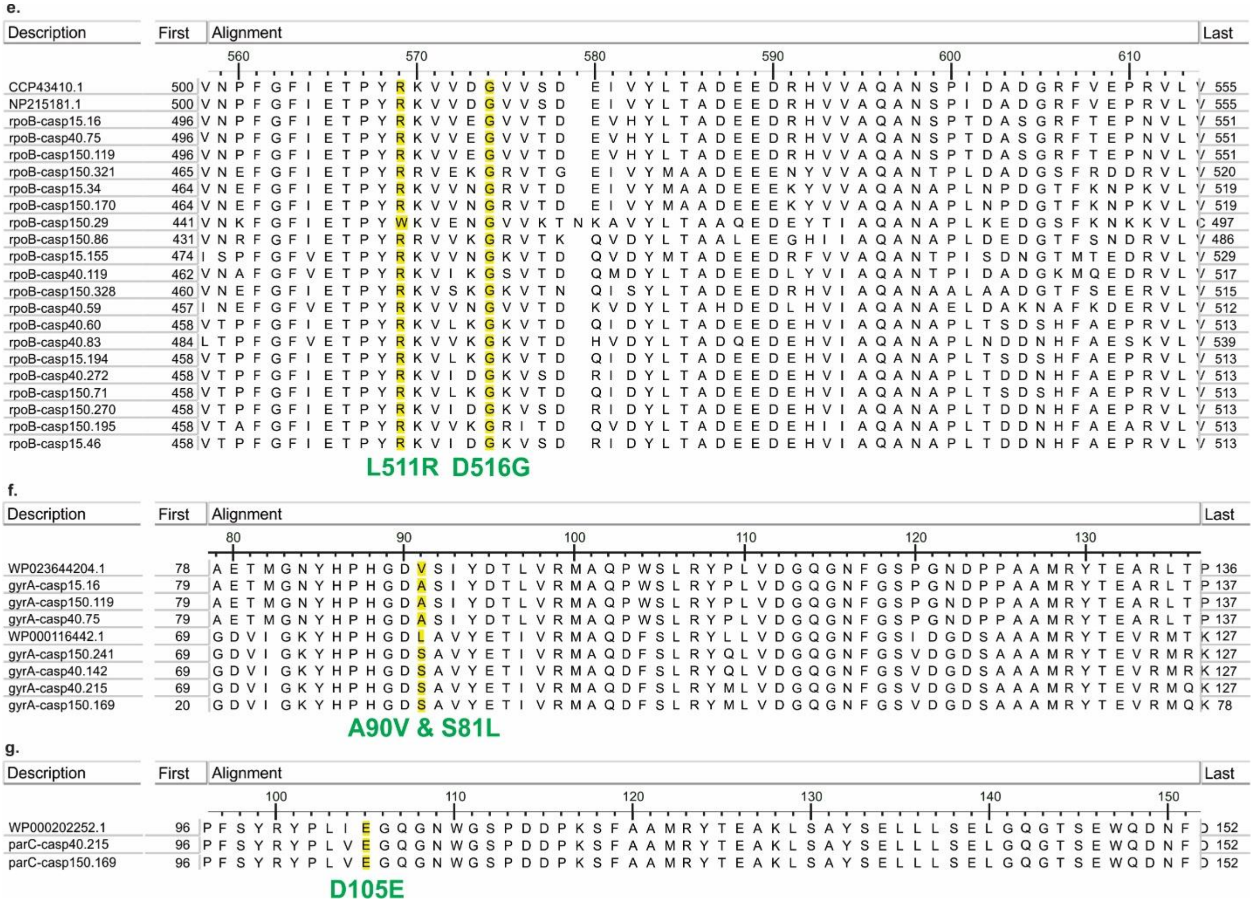

Figure S2. Multiple sequence alignment shows the point mutations that confer the antibiotic resistance. Sequences containing the relevant mutation were confirmed as antibiotic resistance genes. All alignments results are accompanying this manuscript as **Supplementary Data S2**. Reference genes belong to (**a,b,d,e,f**) *Mycobacterium tuberculosis* (WP_003910993, WP_016810233, CCP44394, CCE36834, CCP43410, NP_215181, and WP_023644204) and (**c,f,g**) *Acinetobacter baumannii* (AJF83452, WP_000116442 and WP_000202252). Alignments figures were obtained with the NCBI Genome Workbench v3.7.1.

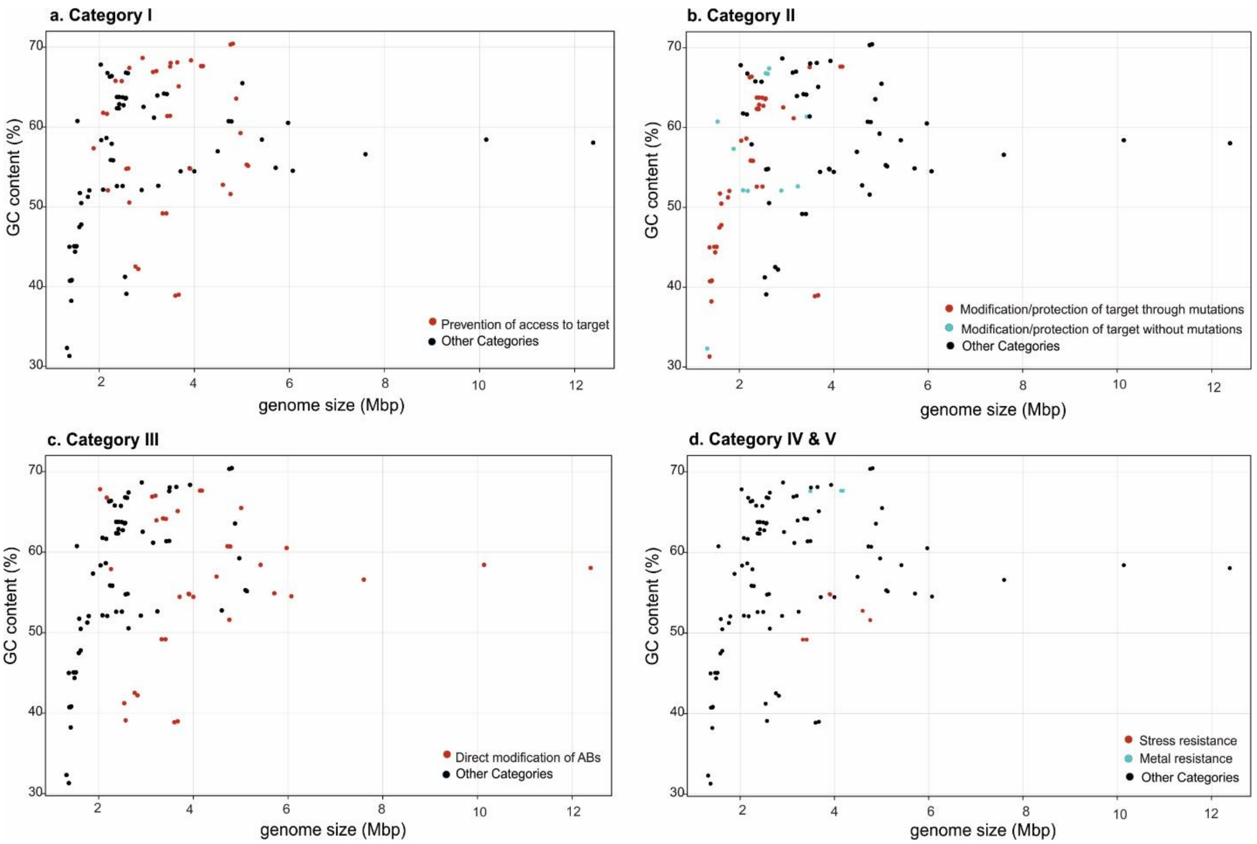

Figure S3. Genomic GC content versus estimated genome size for all ARG containing MAGs. the ARG categories are marked in each plot. Genomic GC content versus estimated genome size are colored based on: (a) Category I, (b)category II (red dots indicate genomes that confer resistant through mutations, which have lower GC content and estimated genome size. Blue dots indicate genomes that confer resistant without mutation.), (c) category III and (d) category IV and V.

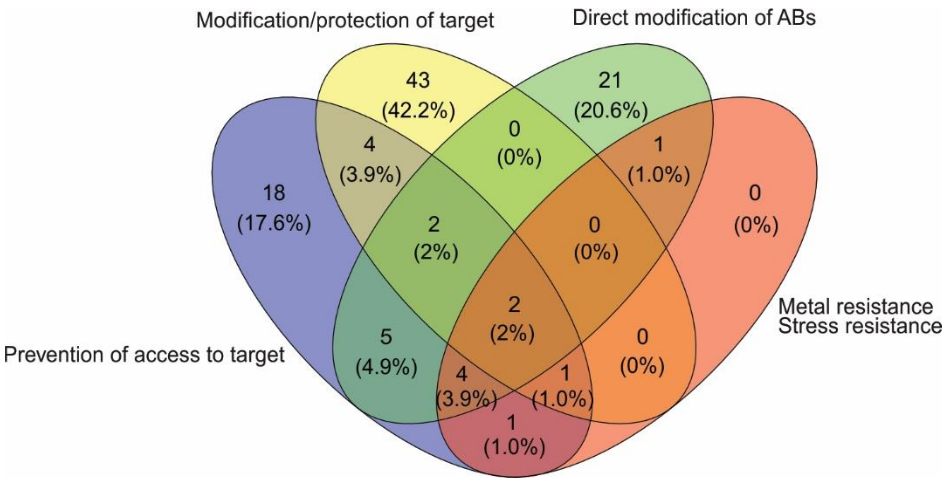

Figure S4. Venn diagram of antibiotic resistance categories. This picture indicates the number of Caspian ARG containing MAGs for each ARG category. 2 MAGs are shared in all the groups and 2 MAGs are shared in three main categories, meaning that these MAGs have various ARGs from diverse categories. Due to the small number of genes in the two categories of Metal resistance and Stress resistance, and also to avoid complicating the Venn diagram, we have considered these two categories as one group.

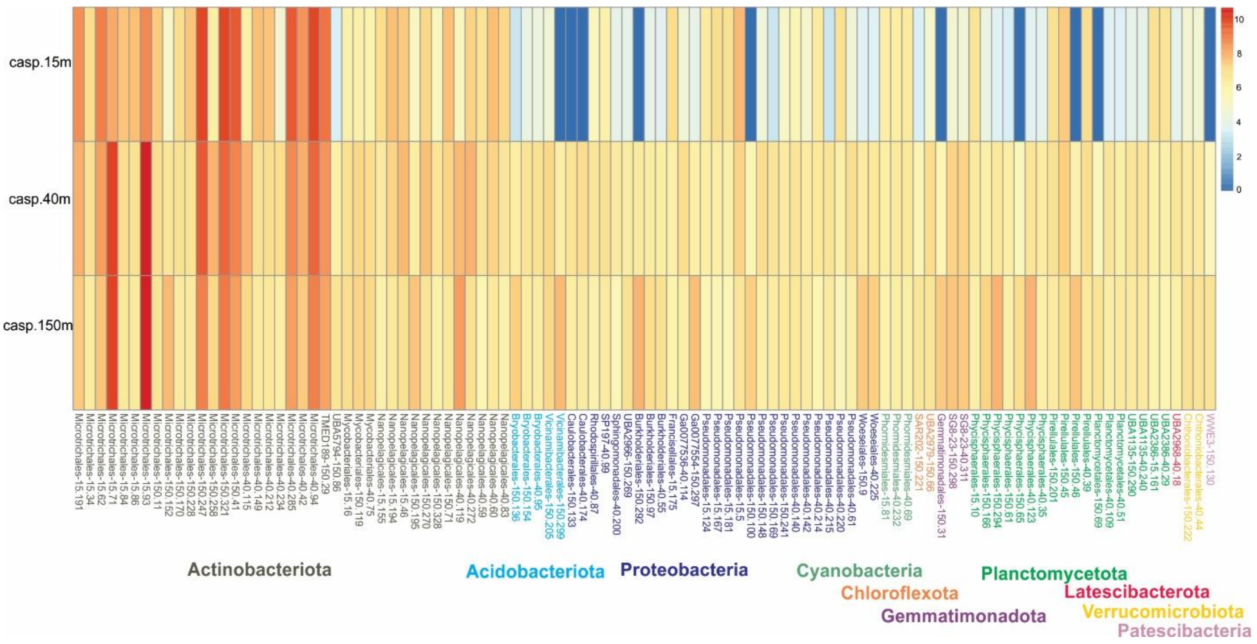

Figure S5. Heat map representation of genome abundance of ARG containing MAGs in different depth of the Caspian Sea. Heat map is drawn in logarithmic scale for better visualizing differences. The members of each phylum are put together for better comparison. Representatives of the class Actinobacteriota have the highest abundance in three depth of the Caspian Sea.

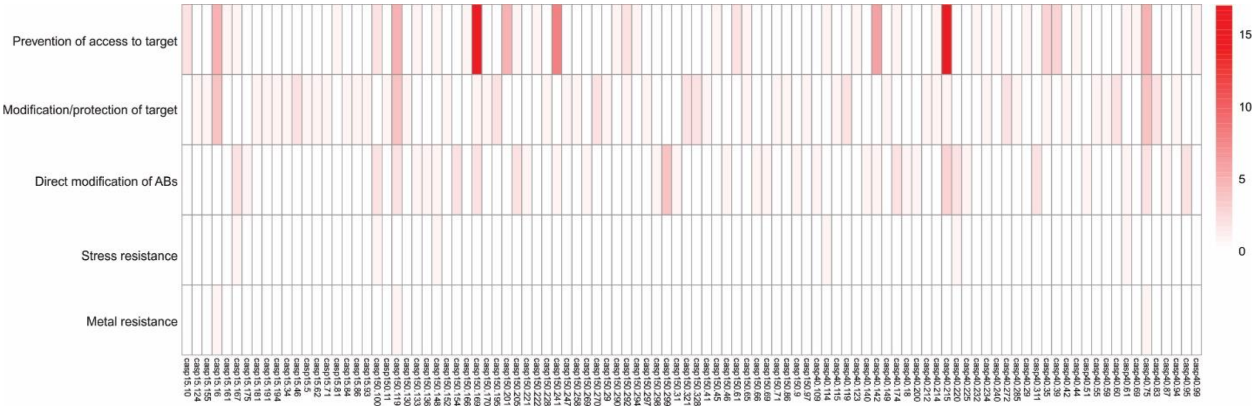

Figure S6. Heat map representation of number of observed ARGs in each Caspian ARG containing MAG with respect to different categories of antibiotic resistance.

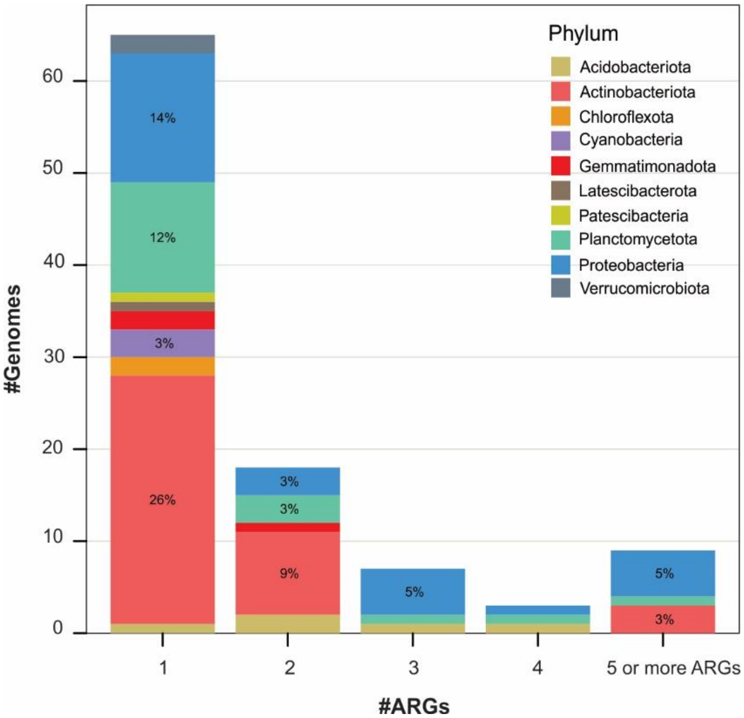

Figure S7. Prevalence of MAGs resistant to one, two, three, four or multiple categories of ARGs with respect to their taxonomic assignment.

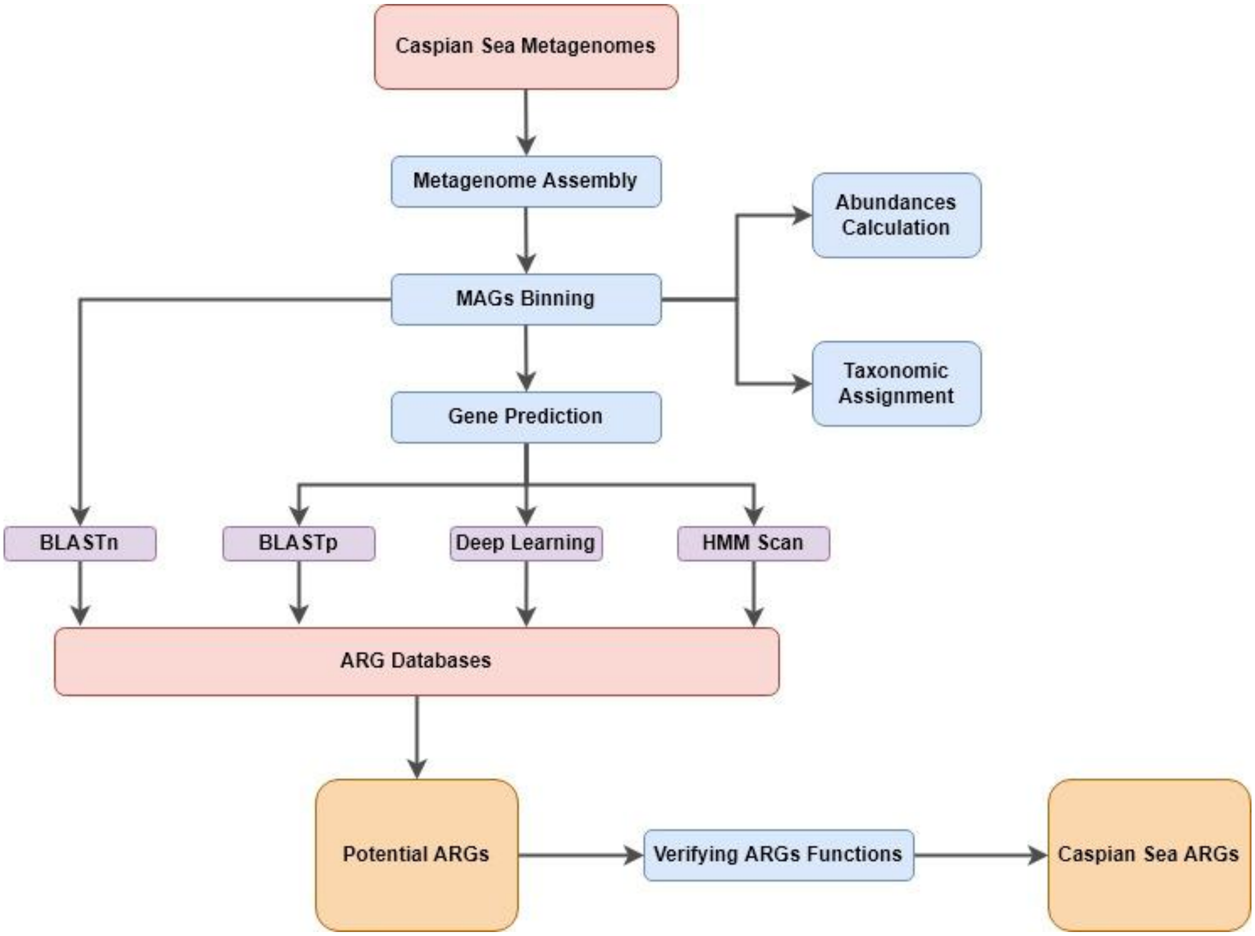

Figure S8. A pipeline workflow diagram describing the steps involved in the Caspian Sea ARGs identification. The red boxes denote inputs, blue boxes represent steps in the study, purple boxes represent the approaches, yellow boxes denote outputs and arrows show the directionality of the workflow.
